# Bruchid beetle ovipositioning mediated defense responses in black gram pods

**DOI:** 10.1186/s12870-020-02796-4

**Published:** 2021-01-11

**Authors:** Debajit Das, Indrani K. Baruah, Debashis Panda, Ricky Raj Paswan, Sumita Acharjee, Bidyut Kumar Sarmah

**Affiliations:** 1 Department of Agricultural Biotechnology, Assam Agricultural University, Jorhat, Assam India; 2 Office of the ICAR-National Professor (Norman Borlaug Chair) and DBT-AAU Centre, Assam Agricultural University, Jorhat, 785013 India; 3 Distributed Information Centre, Department of Agricultural Biotechnology, Assam Agricultural University, Jorhat, Assam India

**Keywords:** Bruchid, Oviposition, *Vigna mungo*, Transcriptome, de novo assembly, *Callosobruchus sp*, Illumina sequencing

## Abstract

**Background:**

Black gram [*Vigna mungo* (L)] seeds are a rich source of digestible protein and dietary fibre, both for human and animal consumption. However, the quality and quantity of the *Vigna* seeds are severely affected by bruchid beetles during storage. Therefore, analyses of the expression of the bruchid induced transcript dynamics in black gram pods would be helpful to understand the underlying defense mechanism against bruchid oviposition.

**Results:**

We used the RNAseq approach to survey the changes in transcript profile in the developing seeds of a moderately resistant cultivar IC-8219 against bruchid oviposition using a susceptible cultivar T-9 as a control. A total of 96,084,600 and 99,532,488 clean reads were generated from eight (4 each) samples of IC-8219 and T-9 cultivar, respectively. Based on the BLASTX search against the NR database, 32,584 CDSs were generated of which 31,817 CDSs were significantly similar to *Vigna radiata*, a close relative of *Vigna mungo*. The IC-8219 cultivar had 630 significantly differentially expressed genes (DEGs) of which 304 and 326 genes up and down-regulated, respectively. However, in the T-9 cultivar, only 168 DEGs were identified of which 142 and 26 genes up and down-regulated, respectively. The expression analyses of 10 DEGs by qPCR confirmed the accuracy of the RNA-Seq data. Gene Ontology and KEGG pathway analyses helped us to better understand the role of these DEGs in oviposition mediated defense response of black gram. In both the cultivars, the most significant transcriptomic changes in response to the oviposition were related to the induction of defense response genes, transcription factors, secondary metabolites, enzyme inhibitors, and signal transduction pathways. It appears that the bruchid ovipositioning mediated defense response in black gram is induced by SA signaling pathways and defense genes such as defensin, genes for secondary metabolites, and enzyme inhibitors could be potential candidates for resistance to bruchids.

**Conclusion:**

We generated a transcript profile of immature black gram pods upon bruchid ovipositioning by de novo assembly and studied the underlying defense mechanism of a moderately resistant cultivar.

**Supplementary Information:**

The online version contains supplementary material available at 10.1186/s12870-020-02796-4.

## Background

Plants possess countless defense mechanisms against insect herbivores to avoid the yield penalty. These inherent mechanisms are either expressed constitutively during plant growth and development or induced upon insect damage. Plants not only responds to adult insects or herbivores but also when the female lays eggs on the plant surface. This oviposition response is often quick to protect from future damage by emerging larvae, although the nature of oviposition associated cues is scarce [1, 2]. The egg induced responses are generally a hypersensitive reaction (HR). Also, several studies revealed a direct plant defense against insect oviposition, including the growth of neoplastic tissues [3, 4] and secretion of ovicidal compounds to kill the eggs [1, 5]. In the case of indirect defense responses, egg deposition results in the emission of volatile compounds known as Oviposition Induced Plant Volatiles (OIPV) to attract egg parasitoids [6–9]. Plants respond to egg deposition by changing the leaf surface chemistry or odor to retain egg parasitoids on leaves [10, 11]. The most preliminary response to the egg deposition on the plant surface is the generation of reactive oxygen species (ROS) [5] followed by the formation of callose [12–14] and the death of plant cells.

Bruchids are notorious stored grain pests of many legumes, including black gram. The pest arrives in the field during the pod formation stage and females oviposit on the surface of the pod wall. However, a rapid multiplication occurs when the harvested seeds are stored. In the field, the plants are sprayed with insecticides to avoid bruchid infestation and harvested seeds to be used as planting materials are also treated with insecticides to prevent bruchid multiplication. Losses are mostly unavoidable because seeds marketed for human consumption are not coated with pesticides. Since female bruchids lay eggs on the pod wall; therefore, how black gram defends against bruchids egg-laying on the pods is vital to understand the resistance mechanism.

The insect egg-mediated plant defense responses are triggered mostly due to the elicitors present in the exocrine secretion which covers the eggs to prevent falling-off and desiccation. The chemical composition of the egg elicitor from adult bruchid weevil (*Bruchus pisorum*) is known as “bruchin”. The active molecule of the bruchin is a C_22_-C_24_ long-chain α, ω-diols esterified with one or both ends occupied by 3-hydroxypropanoic acids. Bruchin is known to elevate the levels of defense responsive phytohormones such as jasmonic acid (JA), salicylic acid (SA), and ethylene (ET) [3, 15], which subsequently, up-regulates defense responsive genes.

Black gram is an important pulse crop grown in tropical and sub-tropical regions of India for its highly nutritious seeds, which are a good source of carbohydrate (60%) and digestible protein (24%) [16]. Bruchid infestation during storage conditions causes significant damage within a period of three to six months, reducing the market value (both quality and quantity) of the seeds [17, 18]. A few cutlivated and wild relatives of black gram have shown moderate resistance to bruchids; however, cross incompatibility impeded the breeding for resistance [19]. The cultivated lines having moderate levels of resistance [17] could be a suitable source to gain an insight into the defense responses or identify the resistance gene.

We studied the oviposition mediated transcriptome changes in black gram to identify defense responsive genes. Previously, we adopted a forward suppression subtractive hybridization (SSH) approach to obtain an overview of the ovipositioning-mediated defense response in black gram. Our SSH libraries unfolded up-regulation of several defense-responsive genes such as defensin, pathogenesis-related protein (PR), receptor serine-threonine kinase (RSTK), dehydration responsive element transcription factor (DRE), heat shock protein 70 (Hsp70) [20]. Our SSH libraries yielded a small representation of differentially expressed genes; therefore, we opted for a transcriptome approach in the current study to get a comprehensive overview of bruchid oviposition-mediated defense response in black gram. The comparative transcriptome sequencing from bruchid oviposited moderately resistant and susceptible cultivars revealed significant up-regulation of several defense responsive genes, transcription factors, secondary metabolites, protein kinases/phosphatases, hormone signalling, and regulation pathways.

## Results

### Oviposition-mediated reactive oxygen species (ROS) generation in black gram pods

**Fig. 1:**
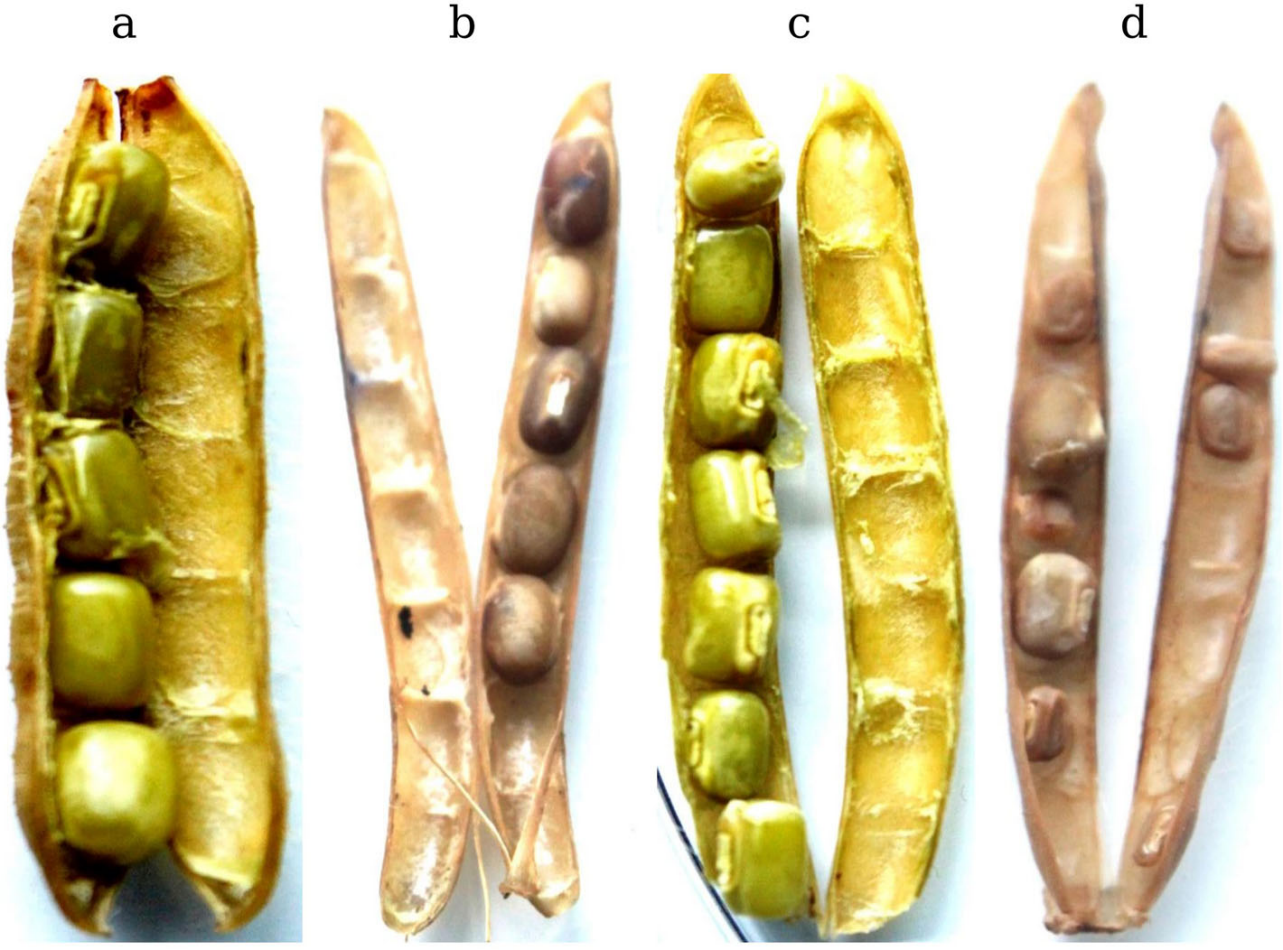
Accumulation of ROS in *Vigna mungo* pods in response to bruchid beetle ovipositioning. **a** Control pod of IC-8219 cultivar. **b** Oviposited pod of IC-8219 cultivar after 7 days. **c** Control pod of T-9 cultivar. **d** Oviposited pod of T-9 cultivar after 7 days

Ovipoistioning by bruchids on the black gram pod showed a hypersensitive response (HR) on the pod which was visible only after removing the eggs from the surface. The HR could generate an oxidative burst by producing reactive oxygen species (ROS), superoxide anions, hydrogen peroxide, hydroxyl radicals, and nitrous oxide. Rapid accumulation of ROS at the site of insect infestation triggers the expression of several pathogenesis-related genes [21]. Therefore, we assayed the generation of ROS upon ovipositioning by bruchids on black gram pods by using a 3, 3- diaminobenzidine (DAB) staining method. The production of hydrogen peroxide (H_2_O_2_) was observed as dark brown discoloration on the oviposited pods of both the cultivars of black gram compared to controls (Fig. 1a, b, c, and d). This HR response through ROS generation indicated bruchid oviposition had triggered the defense responses in the treated samples.

### Sequencing and assembly of transcript sequences of bruchid oviposited black gram pods

To understand the underlying defense mechanism of black gram against bruchid oviposition, cDNA libraries were generated using the total RNA extracted from the immature developing seeds of controls and treated pods of both the cultivars. Paired-end sequencing was done using the NextSeq 500 platform. After removal of low quality reads of < 20 Phred score, a total of 44,880,659 and 51,203,941 clean reads (total length 13,240,367,400 and 15,035,372,861 bp) were obtained for the IC-8219 (C) and IC-8219 (T), respectively, while 51,617,299 and 47,915,189 clean reads (total lengths 15,177,630,537 and 14,054,183,123 bp) were generated for the T-9 (C) and T-9 (T) samples, respectively (Table 1).

**Table 1:** Summary of high quality reads of *V. mungo* transcriptome obtained from pods of IC-8219 and T-9 cultivars after 7 days of ovipositioning (T) by bruchids along with non-oviposited(C) pods

Sample	Number of paired end reads	Size of paired end reads (bp)
IC-8219 (T)	26,838,337	7,907,548,094
IC-8219 (T)	24,365,604	7,127,824,767
IC-8219 (C)	24,799,219	7,292,871,647
IC-8219 (C)	20,081,440	5,947,495,753
T-9 (T)	25,245,800	7,402,238,660
T-9 (T)	22,669,389	6,651,944,468
T-9 (C)	21,566,510	6,359,937,646
T-9 (C)	30,050,789	8,817,692,891

**Fig. 2:**
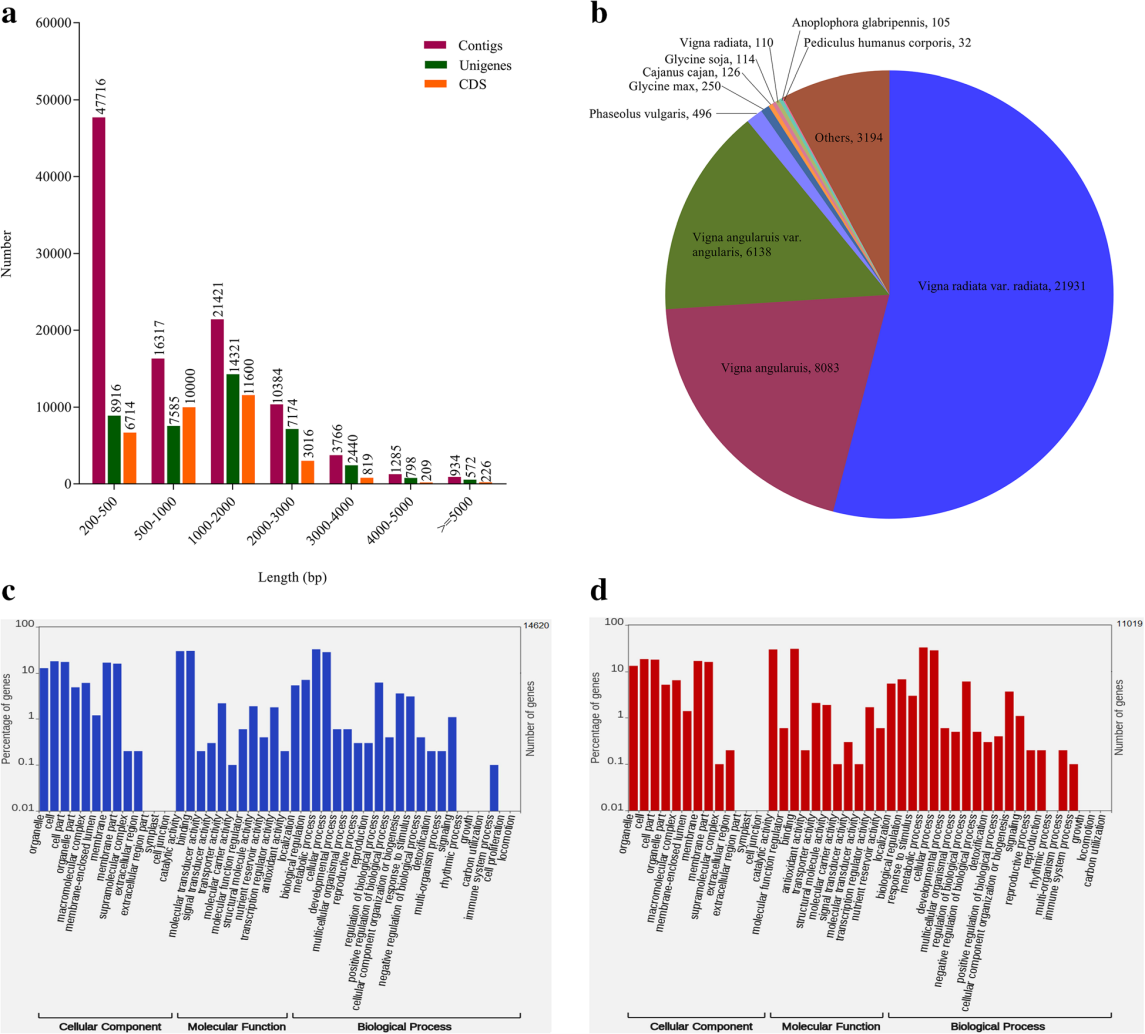
Overview of *Vigna mungo* transcript assembly. **a** Length distribution of contigs, unigenes, and CDSs. **b** Species distribution of top BLAST hits of assembled CDSs (E value ≤10^− 5^). **c** Functional annotation of CDSs based on GO categorization in the IC-8219 cultivar. **d** Functional annotation of CDSs based on GO categorization in the T-9 cultivar

The whole-genome sequencing of black gram is now available but poorly annotated [22]; therefore, the raw reads were assembled by the Trinity de novo software for RNAseq data. The raw reads were also aligned with the available black gram genome sequence and found to be > 90% similar (Additional Fig. 1). The clean reads were assembled into 101,823 contigs with reads length of which ranged from 201 to 15,724 bp with a mean length of 1057 bp having an N50 value of 1887 bp. A total of 47,716 (46.86%), 16,317 (16.02%) and 21,421 (21.04%) contigs were found to be in the range of 200–500 bp, 500–1000 bp and 1000–2000 bp, respectively. The contigs were further assembled using the CD-HIT-EST-4.5.4 into a total of 41,806 unigenes with an average length of 1498 bp and an N50 value of 2078 bp (Table 2). Among all the unigenes, 8916 (21.33%), 7585 (18.14%) and 14,321 (34.26%) were in the range of 200–500 bp, 500–1000 bp and 1000–2000 bp, respectively. In all, we obtained a total of 32,584 CDSs of which 6714 (20.61%), 10,000 (30.69) and 11,600 (35.60%) were in the range of 200–500 bp, 500–1000 bp, and 1000–2000 bp, respectively. Since the coding sequences were of high-quality; therefore, downstream analyses were performed. The length distribution of contigs, unigenes, and coding sequence is shown in (Fig. 2a). A total of 22,993, 14,620, 29,542, and 11,019 CDSs were identified for IC-8219 (C), IC-8219 (T), T-9 (C), and T-9 (T) samples, respectively, from the pooled set of CDS with a minimum CDS length of 297 bp for all the samples. The maximum CDS length of 15,444 bp was recorded in the control samples, while 12,714 bp was the maximum length of CDS for the treated samples (Table 3).

**Table 2:** Summary of the Illumina paired end sequencing and de novo assembly for *V. mungo* transcripts

Database	Number
Total clean reads	195,617,088
Total length of clean reads (bp)	57,507,553,926
Assembly	Trinity
Number of contigs	101,823
Total length of contigs (bp)	107,355,854
Average length of contigs (bp)	1054
Max length of contigs (bp)	15,724
Min length of contigs (bp)	201
Contig size N50 (bp)	1887
Number of unigenes	41,806
Total length of unigenes (bp)	62,639,845
Average length of unigenes (bp)	1498
Max length of unigenes (bp)	15,724
Min length of unigenes (bp)	201
Unigene size N50 (bp)	2078

**Table 3:** Sample wise CDS statistics

Description	IC-8219 (C)	IC-8219 (T)	T-9 (C)	T-9 (T)
No. of CDS	29,542	11,019	22,993	14,620
Total CDS length (bases)	35,972,616	12,191,925	28,428,723	16,215,216
Maximum CDS length	15,444	12,714	15,444	12,714
Minimum CDS length	297	297	297	297
Mean CDS length	1217	1106	1236	1109

### Annotation and classification of oviposition-induced coding sequences

To obtain complete functional annotation, all the assembled CDSs were aligned against the non-redundant (NR) protein database from the NCBI using the BLASTx program (E value ≤10^− 5^). Out of 32,584 annotated CDSs, 31,817 (97.65%) of the annotated CDSs were aligned to the NR database, while 767 (2.35%) CDSs were not represented in the blast hits. The homology search result showed that the majority of the blast hits were from *V. radiata* (21,931). Mungbean (*V. radiata*) is a close relative of black gram, therefore, a high homology of black gram transcript was expected. The remaining CDSs showed similarity with sequences of *V. angularis* (8083) followed by *V. angularis* var. *angularis* (6138) as represented in Fig. 2b. The low representation of hits with *V. mungo* indicated that little information of its genome sequence is available in the public database.

Based on the NR annotation, a total of 9169 and 7066 assembled CDSs from IC-8219 (T) and T-9 (T) samples, respectively, were subjected to the gene ontology (GO) classification using the Blast2Go. In all, 5879, 4469 and 7186 CDSs from IC-8219 (T) and 4491, 3428 and 5471 CDSs from T-9 (T) sample were classified into 3 major domains, biological process, cellular component and molecular function, respectively. The DEGs of IC-8219 cultivar were divided into a total of 46 sub-categories of which 20, 13, and 13 sub-categories belonged to biological process, molecular function, and cellular component, respectively (Fig. 2c). The DEGs of T-9 cultivar were divided into 45 sub-categories with 20, 12, and 13 sub-categories in biological processes, cellular component, and molecular function, respectively (Fig. 2d). The most abundant subcategories of the classified genes include “metabolic process” and “cellular process” under the category of biological process. Similarly, “cell”, “cell part”, “membrane” and “membrane part” were subcategories under the cellular component, while subcategories, “catalytic activity”, and “binding” were grouped under the molecular function for both IC-8219 and T-9 cultivar.

**Fig. 3:**
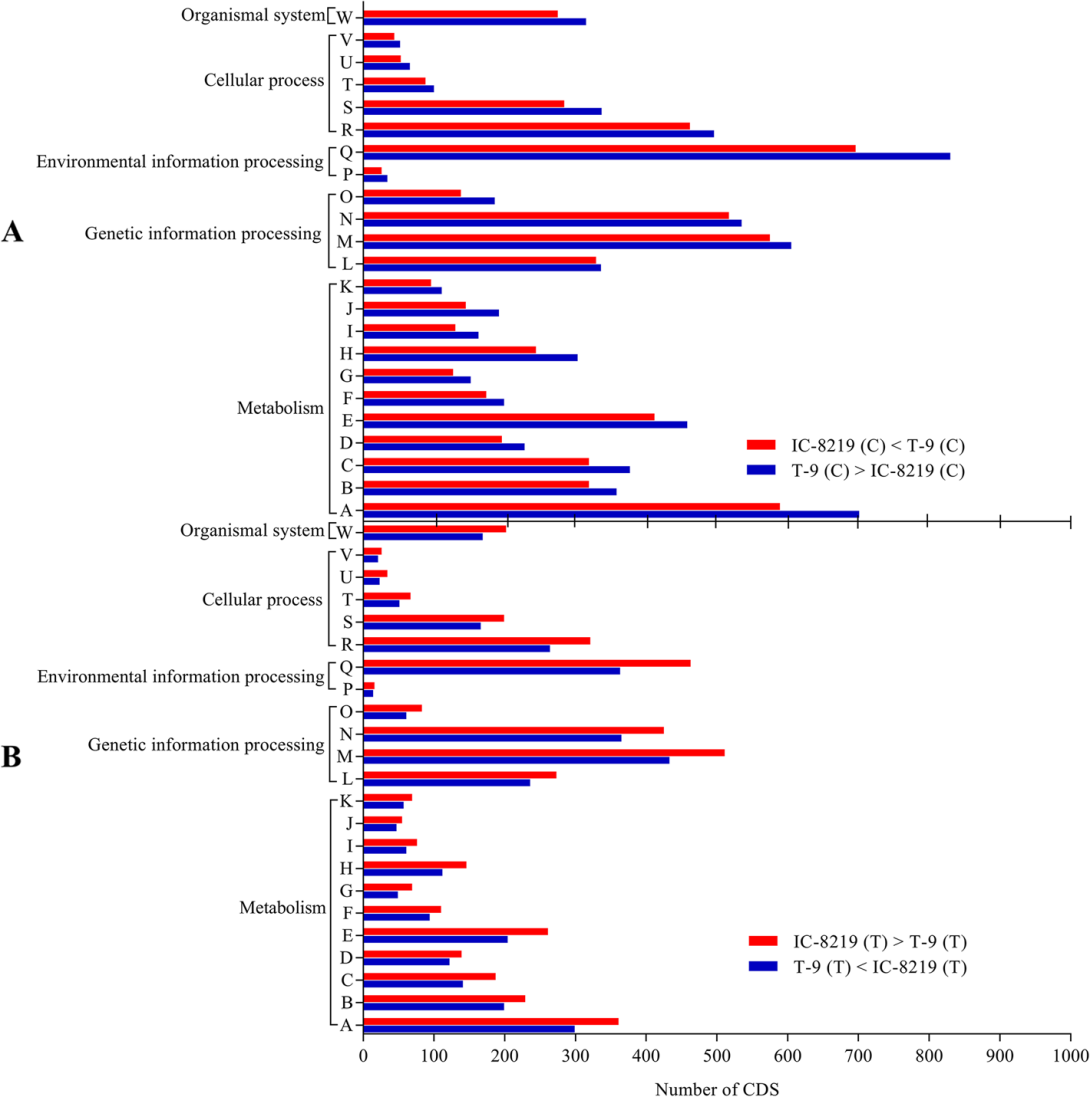
Classification of CDSs based on the KEGG pathway of IC-8219 (C), IC-8219 (T), T-9 (C), and T-9 (T) samples. A total of 6238 and 7134 CDSs from IC-8219 (C) and T-9 (C) samples (**a**) and 4322 and 3551 CDSs from IC-8219 (T) and T-9 (T) samples (**b**) were classified into 23 KEGG pathways

To identify the potential biological pathways in *V. mungo,* the pathway annotation of the predicted CDSs was performed using the Kyoto Encyclopedia of Genes and Genomes (KEGG) [23]. The KEGG pathway annotated 4322 and 3551 CDSs from IC-8219 (T) (Additional file 1: Table S1) and T-9 (T) (Additional file 1: Table S1) samples, respectively, into 23 KEGG pathways under five major categories, including metabolism, genetic information processing, environmental information processing, cellular processes, and organismal systems. Among the 23 identified KEGG pathways, the largest group of clusters in the treated samples of IC-8219 and T-9 cultivar under the genetic information processing category were “translation” (511 CDS of IC-8219 and 433 CDS of T-9) and “folding, sorting and degradation” (425 CDS of IC-8219 and 365 CDS of T-9) and under the environmental information processing was sub-categorized as “signal transduction” (463 CDS of IC-8219 and 363 CDS of T-9). Similarly, under the metabolism category, the “carbohydrate metabolism” (361 CDS of IC-8219 and 299 CDS of T-9) was the dominant pathway, while under the cellular process the major pathway was “Transport and Catabolism” (321 CDS of IC-8219 and 264 CDS of T-9). The observations were depicted in Fig. 3 and incorporated in Table S2 of Additional file 2.

### DEGs of bruchid oviposited black gram

**Fig. 4:**
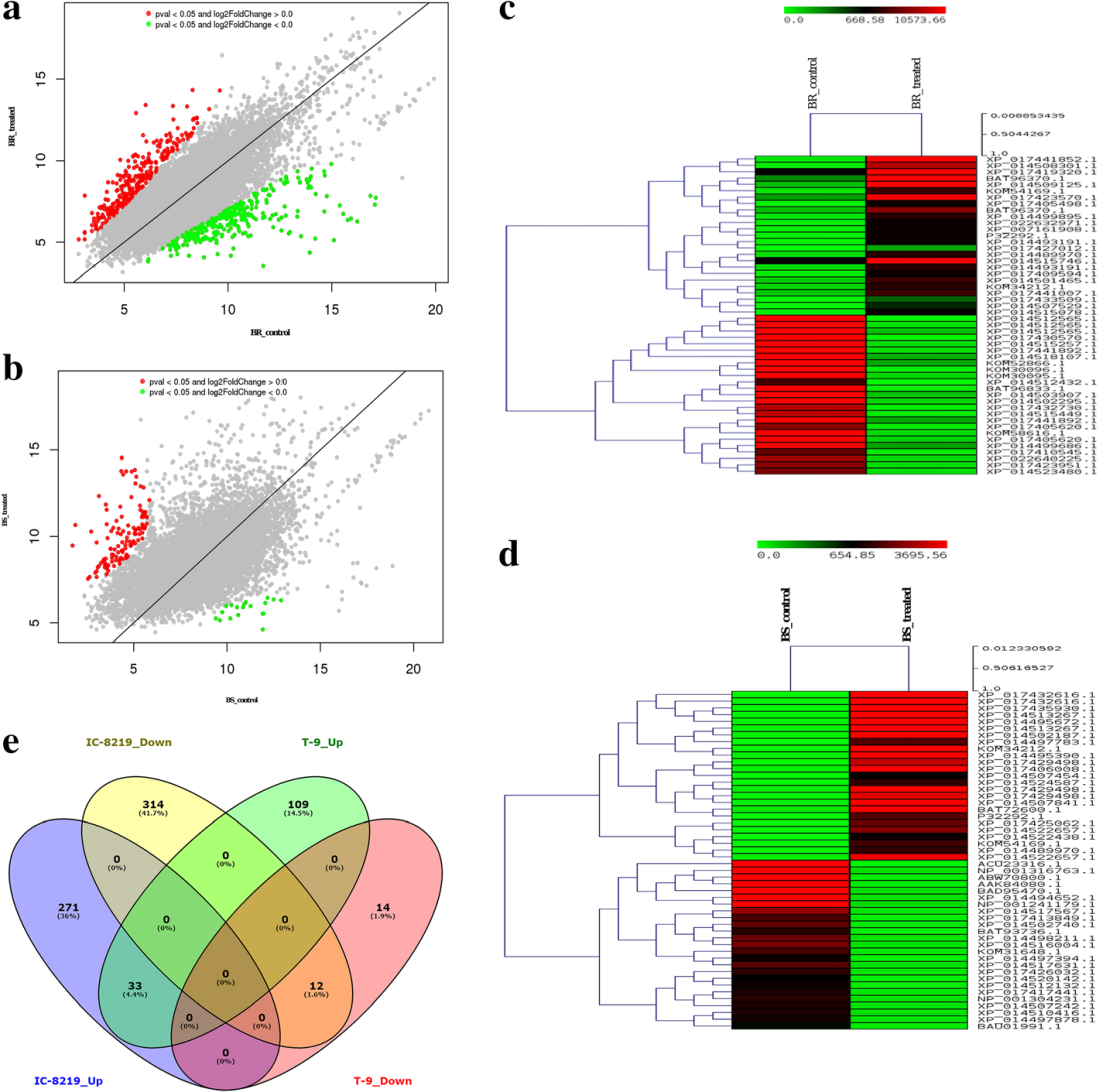
Overview of DEGs expressed in both the cultivars of black gram in response to bruchid beetle ovipositioning on black gram pods. **a** Scatter plot of DEGs in IC-8129. **b** Scatter plot of DEGs in T-9. **c** Heat map of top 50 DEGs in IC-8129. **d** Heat map of top 50 DEGs in T-9. **e** Venn diagram showing the commonly expressed genes between IC-8219 and T-9 during bruchid oviposition. *BR indicates the moderately resistant cultivar, BS indicates susceptible cultivar

To obtain a comprehensive understanding of the transcript expression of black gram in response to bruchid oviposition, we identified several genes that were differentially expressed between the control samples and oviposited samples. The transcript dynamics of the ovipoisted samples of both IC-8219 and T-9 cultivar were compared with the control samples and represented in scatter plots (Fig. 4a and b). A large set of genes were differentially expressed in the IC-8219 cultivar (Additional file 3: Table S3) and T-9 cultivar (Additional file 3: Table S3). In all, 630 significant DEGs were identified in the IC-8219 cultivar using the DESeq software [24]. Among these DEGs, 304 genes were up-regulated and 326 genes down-regulated compared to the non-oviposited control (within the cultivar). For the T-9 cultivar, 168 DEGs were detected of which 142 genes up-regulated and 26 genes down-regulated compared to the non-oviposited control (within the cultivars). The results depicted in heat maps showed that the number of DEGs is more in the IC-8219 cultivar compared to the T-9 cultivar (Fig. 4c and d). Compared with the S (T) sample, 539 DEGs were identified of which 169 genes up- and 370 genes down-regulated in the IC-8219 (T) sample (between the cultivar). Thus the IC-8219 cultivar showed 169 up-regulated and 370 down-regulated genes when compared with T-9. In all, 45 DEGs were shared by both IC-8219 and T-9 cultivars of which 33 were up-regulated and 12 were down-regulated (Fig. 4e).

### GO enrichment of DEGs

The functional categories of the DEGs in the IC-8219 and T-9 cultivars induced due to bruchid oviposition were obtained through GO enrichment. In IC-8219, the most significantly enriched GO category was “metabolic process“ followed by “oxidation-reduction process”, “signal transduction”, “oxidoreductase activity, “nucleus”, “regulation of transcription”, “proteolysis”, “hydrolase activity”, “transferase activity”, “transmembrane transport“ and “response to stress” (Additional file 4: Table S4). Whereas, in T-9 the dominant GO category was “oxidation-reduction process” followed by “oxidoreductase activity”, “metabolic process”, “regulation of transcription”, “mitochondrion”, “response to stress”, “chloroplast”, “transferase activity”, “nucleus”, “signal transduction” and “transmembrane transport” (Additional file 4: Table S4). Based on our data it appears that bruchid oviposition-mediated defense response in black gram is a complex process.

### Pathway enrichment analysis of DEGs

The biological pathways of DEGs which showed significant changes in oviposited black gram pods were identified using the KEGG database. Pathway analysis revealed 23 biological pathways of which 10 pathways were common between IC-8219 and T-9 cultivar. The common pathways found between these two cultivars were mostly associated with carbohydrate metabolism (ko00051, ko00520), energy metabolism (ko00196, ko00920), nucleotide metabolism (ko00230), biosynthesis of other secondary metabolites (ko00943), translation (ko03010), folding, sorting and degradation (ko03060, ko04141) and signal transduction (ko04016) (Additional file 5: Table S5). In total, 23 DEGs in the IC-8219 cultivar showed involvement in carbohydrate metabolism, of which 22 DEGs were found to be down-regulated. Contrastingly, 10 DEGs associated with the energy metabolism were significantly induced in the T-9 cultivar. DEGs involving in sulfur metabolism (ko00920), isoflavonoid biosynthesis (ko00943), and MAPK signaling pathway-plant (ko04016) showed significant fold change in both the cultivars. DEGs in the IC-8219 cultivar associated with lipid metabolism were significantly down-regulated (ko00071, ko00073, ko00100, ko00591, ko00592) except two DEGs (ko00564, ko00600) which were induced upon oviposition. The repressed DEGs of lipid metabolism were related to oxylipin biosynthesis and jasmonic acid-mediated signaling cascade.

### Differential expression of defense-related genes

Bruchid ovipositioning resulted in differential expression of 43 (23 induced and 20 repressed) and 11 (10 induced and 1 repressed) defense-related genes in IC-8219 (Table 4) and T-9 cultivar (Additional file 6: Table S6), respectively. These DEGs of defense genes in IC-8219 are grouped as 6 (4 induced and 2 repressed) classical salicylic acid-responsive marker genes such as PR2, thaumatin, glucan-endo-1,3-ß-glucosidase, and other PR genes which could have accumulated in black gram pods during the HR. Based on our data these 4 induced genes showed a fold change of > 3 in IC-8219. The majority (9) of defense genes found in IC-8219 were associated with post-transcriptional regulation, of which 8 genes were induced due to ovipositioning. These 8 genes encoded the pentatricopeptide repeat-containing protein (PPR) having distinctive roles in RNA metabolism. Interestingly, 14 defense genes (6 induced and 8 repressed) having protease activity were highly represented in the IC-8219 cultivar. Also, one DEG of aspartic protease activity (BAT85009.1) was induced in both the cultivar. Two genes of an enzyme encoding nudix hydrolase were either induced or repressed in IC-8219. We also observed transcriptional up-regulation of SA dependent pathogen-induced defense genes, LURP-one-related 8, in the IC-8219 cultivar.

**Table 4:** Differentially expressed defense-related genes identified in the IC-8219 cultivar due to bruchid ovipositioning

Gene ID	Annotation	log_2_ Fold Change	FDR (≤0.05)
XP_014516032.1	Protein LURP-one-related 8-like	3.22	0.0049
XP_014509125.1	Pathogenesis-related protein 2-like	6.02	0.0051
XP_014516032.1	Protein LURP-one-related 8-like	3.77	0.0151
XP_014502683.1	Glucan endo-1,3-beta-glucosidase	3.68	0.0183
BAT85009.1	Hypothetical protein VIGAN_04249700	4.31	0.0030
BAT85009.1	Hypothetical protein VIGAN_04249700	4.40	0.0035
BAT85009.1	Hypothetical protein VIGAN_04249700	4.24	0.0082
BAT85009.1	Hypothetical protein VIGAN_04249700	3.97	0.0155
XP_014504733.1	Universal stress protein A-like protein	2.96	0.0189
XP_014520780.1	Thaumatin-like protein	3.58	0.0202
XP_014517700.1	Protease Do-like 5	2.63	0.0337
XP_014514844.1	Pentatricopeptide repeat-containing protein At1g62350	2.56	0.0365
XP_007155737.1	Hypothetical protein PHAVU_003G2274001g	2.31	0.0396
XP_014496563.1	Pentatricopeptide repeat-containing protein At3g29230	2.64	0.0401
XP_014493692.1	Pentatricopeptide repeat-containing protein At1g05600	2.55	0.0433
XP_014496563.1	Pentatricopeptide repeat-containing protein At3g29230	2.53	0.0454
XP_014514844.1	Pentatricopeptide repeat-containing protein At1g62350	3.08	0.0123
XP_014514844.1	Pentatricopeptide repeat-containing protein At1g62350	2.72	0.0148
XP_014514844.1	Pentatricopeptide repeat-containing protein At1g62350	2.97	0.0206
XP_014491746.1	Pentatricopeptide repeat-containing protein At1g06270	2.42	0.0485
XP_017421074.1	Pathogen-related protein	3.15	0.0487
NP_001304240.1	Putative threonine aspartase	2.38	0.0484
XP_014517990.1	Nudix hydrolase 15	3.34	0.0026
XP_014523480.1	P34 probable thiol protease	−5.97	0.0005
XP_017432730.1	Albumin-1-like	−7.40	0.0008
XP_014502603.1	Probable isoaspartyl peptidase/L-asparaginase 2	−5.62	0.0023
XP_014523480.1	P34 probable thiol protease	−6.06	0.0028
XP_017432243.1	Acidic endochitinase	−5.22	0.0052
XP_014514398.1	Subtilisin-like protease	−5.53	0.0069
XP_014502021.1	Protein ASPARTIC PROTEASE	−3.66	0.0084
XP_014496085.1	Cathepsin B-like	−3.95	0.0141
XP_014502142.1	Thiol protease aleurain-like	−4.44	0.0143
XP_014493578.1	Vicilin-like antimicrobial peptides 2–2	−3.71	0.0222
XP_014496085.1	Cathepsin B-like	−2.87	0.0304
XP_014489680.1	Pentatricopeptide repeat-containing protein At2g30100	−3.44	0.0380
XP_014498257.1	Glucan endo-1,3-beta-glucosidase	−2.93	0.0417
XP_014496087.1	Universal stress protein A-like protein	−2.52	0.0425
BAA76744.1	Asparaginyl endopeptidase	−3.49	0.0330
XP_014500967.1	Nudix hydrolase 3-like	−4.12	0.0212
XP_014500967.1	Nudix hydrolase 3-like	−3.99	0.0451
XP_014508093.1	Snakin-2-like	−3.94	0.0135
XP_014503907.1	Snakin-2-like	−8.13	0.0089
XP_014523088.1	Chymotrypsin inhibitor 3-like	−2.51	0.0327

However, the T-9 cultivar showed differential expression of a few defense transcripts such as endochitinase, PR4 (XP_014492850.1), protein LURP-one-related 17 (XP_014520000.1), universal stress protein (XP_014495383.1), protein downy mildew resistance 6-like (XP_017417297.1), and aspartic protease (BAT85009.1). Also, 3 low temperature-induced proteins (XP_014512922.1, XP_014512923.1, XP_014512922.1) were upregulated in T-9 cultivar.

### Transcription factors in oviposited black gram pods

Transcription factors (TFs) regulate the expression of a large set of downstream genes associated with specific physiological processes and genotype. We found differential expression of 36 (22 induced and 14 repressed) and 13 (11 induced and 2 repressed) TFs in IC-8219 and T-9 cultivar, respectively. The transcriptome analysis revealed that many TFs genes were differentially (> 2 fold) expressed in IC-8219 compared to T-9. In both the cultivars, AP2, ethylene-responsive, zinc finger, NAC, heat stress-responsive TFs families were differentially expressed. About 5 TFs genes (XP_014500160.1, XP_014489970.1, XP_014514818.1 and XP_017417818.1 induced and XP_014494652.1 repressed) were found to be common between IC-8219 and T-9. However, MYB, homeobox-leucine zipper, MAD-box TFs were expressed only in IC-8219. Among the different TFs families of IC-8219, zinc finger proteins were highly represented (8 induced and 2 repressed) followed by AP2-ERF TF (3 induced), ERF transcription factor (2 induced), NAC domain-containing protein (1 induced and 1 repressed), heat stress-responsive protein (2 induced), homeobox leucine zipper (1 induced and 1 repressed), *MYB* (1 induced) and *MAD* box (1 induced) (Table 5). Among the different zinc finger TFs, SNO-regulated gene1 (SRG1) was the most abundant TF in IC-8219.

**Table 5:** Differentially expressed transcription factors found in the IC-8219 cultivar due to bruchid ovipositioning

Gene ID	Annotation	log_2_ Fold Change	FDR (≤0.05)
XP_022632971.1	Ethylene-responsive transcription factor	4.86	0.0020
XP_017409594.1	Zinc finger protein CONSTANS-LIKE 5	4.62	0.0036
XP_014489970.1	Ethylene-responsive transcription factor	4.70	0.0054
XP_014521482.1	Heat stress transcription factor	3.25	0.0137
XP_014509993.1	Homeobox protein knotted-1	3.10	0.0293
XP_014514818.1	AP2-like ethylene-responsive transcription factor	3.48	0.0339
XP_014521482.1	Heat stress transcription factor	3.36	0.0345
XP_014514818.1	AP2-like ethylene-responsive transcription factor	3.42	0.0359
XP_017414060.1	Agamous-like MADS-box protein	2.45	0.0363
XP_014497665.1	Scarecrow-like protein	2.96	0.0380
XP_014498272.1	NAC domain-containing protein	3.22	0.0387
XP_017417818.1	Zinc finger protein ZAT11-like	2.56	0.0419
XP_014507260.1	Zinc finger AN1 domain-containing stress-associated protein	2.38	0.0436
XP_014511935.1	Transcription factor MYB3-like	2.40	0.0444
XP_014495339.1	Transcription initiation factor IIB-2	3.31	0.0446
XP_017419795.1	Homeobox-leucine zipper protein	3.32	0.0452
XP_014514818.1	AP2-like ethylene-responsive transcription factor	3.16	0.0474
XP_014500160.1	Protein SRG1-like	3.13	0.0423
XP_017424133.1	Protein SRG1-like	2.94	0.0192
XP_014500160.1	Protein SRG1-like	3.21	0.0212
XP_014500160.1	Protein SRG1-like	3.18	0.0224
XP_014500160.1	Protein SRG1-like	3.10	0.0260
XP_014502295.1	Dehydration-responsive protein RD22	−7.75	0.0054
XP_014494652.1	Ocs element-binding factor 1-like	−4.27	0.0113
XP_014494652.1	Ocs element-binding factor 1-like	−3.84	0.0179
XP_014505025.1	Nuclear transcription factor Y subunit A-1-like	−3.50	0.0275
XP_014494140.1	General transcription factor IIE subunit 1-like	−3.29	0.0294
XP_014491431.1	C-Myc-binding protein	−2.32	0.0301
XP_014494340.1	Homeobox-leucine zipper protein	−3.60	0.0305
XP_014497765.1	Transcription factor HBP-1b(c38)-like	−3.62	0.0363
XP_014505025.1	Nuclear transcription factor Y subunit A-1-like	−3.16	0.0420
XP_014501476.1	Zinc finger CCCH domain-containing protein	−2.98	0.0442
XP_014505733.1	Transcription factor bHLH82-like isoform X1	−2.78	0.0446
XP_017422931.1	NAC transcription factor	−3.06	0.0466
XP_014498713.1	Zinc finger protein ZAT10-like	−2.86	0.0483
XP_014497162.1	RING-H2 finger protein	−2.44	0.0459

### Differentially expressed genes of phenylpropanoid and oxidative stress pathways

Genes of the phenylpropanoid pathway play a crucial role in plant defense response against biotic stresses [25]. In all, 17 DEGs (14 induced and 3 repressed) associated with the phenylpropanoid pathway were found in the IC-8219 cultivar (Table 6). Amongst these DEGs, caffeoyl-CoA O-methyltransferase showed a log_2_ fold change of > 7 in IC-8219. However, in the T-9 cultivar, only 7 DEGs were found to be related to the phenylpropanoid pathway (Additional file 6: Table S6).

**Table 6:** The list of DEGs obtained in the IC-8219 cultivar which are involving in the phenylpropanoid pathway

Gene ID	Annotation	log_2_ Fold Change	FDR (≤0.05)
XP_014508301.1	Probable caffeoyl-CoA O-methyltransferase	7.37	0.0003
XP_017441852.1	Probable caffeoyl-CoA O-methyltransferase	7.40	0.0005
XP_014521804.1	7-deoxyloganetin glucosyltransferase	3.93	0.0110
XP_017441007.1	Anthranilate N-methyltransferase-like	4.48	0.0109
XP_014515710.1	UDP-glucose flavonoid 3-O-glucosyltransferase	3.83	0.0127
XP_014519945.1	2-hydroxyisoflavanone dehydratase	2.89	0.0145
XP_014516798.1	BAHD acyltransferase DCR	2.68	0.0204
XP_017419759.1	Caffeoylshikimate esterase	3.73	0.0245
XP_017419759.1	Caffeoylshikimate esterase	3.43	0.0285
XP_017440855.1	BAHD acyltransferase DCR	2.67	0.0360
XP_014505411.1	4-hydroxyphenylpyruvate dioxygenase	3.66	0.0391
XP_017419759.1	Caffeoylshikimate esterase	3.46	0.0426
NP_001316736.1	7-deoxyloganetic acid glucosyltransferase	2.12	0.0428
XP_017435211.1	7-deoxyloganetin glucosyltransferase	2.28	0.0467
AHA84274.1	Trans-cinnamate 4-monooxygenase	−3.44	0.0112
XP_017415029.1	Probable methyltransferase	−3.26	0.0290
NP_001304077.1	Trans-cinnamate 4-monooxygenase	−2.83	0.0422

Various metabolic processes of phenypropanoid, alkaloid, and terpenoid pathways are known to regulate by the detoxifying enzyme cytochrome P450 oxidases (CYP450s). We found 6 CYP450 genes (5 induced and 1 repressed) expressing differentially in IC-8219 (Additional file 6: Table S6), while only one CYP450 gene was up-regulated in the T-9 cultivar (Additional file 6: Table S6).

HSPs have also been reported to be involved in plant defense response against wounding. Out of 7 DEGs encoding HSPs, 5 were induced in the IC-8219 cultivar by bruchid ovipositioning and only 2 were repressed (Additional file 6: Table S6). In the T-9 cultivar, the up-regulation of only two DEGs encoding HSPs was observed (Additional file 6: Table S6).

Genes related to cell wall reinforcement were also found to be differentially expressed in both the IC-8219 and T-9 cultivar in response to bruchid oviposition. In the IC-8219 cultivar, 16 DEGs (2 induced and 14 repressed) associated with cell wall remodeling were identified (Additional file 6: Table S6). Whereas, only one up-regulated DEG associated with cell wall modification was detected in the T-9 cultivar (Additional file 6: Table S6).

ROS serves as an important signal to regulate the HR mediated cell death. Plants have developed sophisticated mechanisms to minimize the harmful effect of ROS over-accumulation. We found 16 DEGs (10 induced and 6 repressed) that were associated with ROS detoxification in the IC-8219 cultivar (Additional file 6: Table S6). DEGs encoding cystathionine β-synthase (CBS) domain-containing protein (CDCP) which is an important redox regulator of thioredoxins in the ferredoxin-Trx system was highly (6 induced) represented in IC-8219 followed by ubiquinol oxidase (1 induced), copper chaperone for superoxide dismutase (1 induced), nifU-like protein 4 (1 induced), 1-Cys peroxiredoxin (2 repressed), superoxide dismutase [Fe] (1 repressed), probable glutathione S-transferase (1 repressed), probable peroxygenase (1 repressed) and thioredoxin-like 1–1 (1 repressed). In the T-9 cultivar, 3 DEGs of oxidative stress-related CBS domain-containing protein (1 induced) and probable 2-oxoglutarate/Fe (II)-dependent dioxygenase (2 induced) were differentially expressed (Additional file 6: Table S6).

### Expression profile of genes associated with transcriptional reprogramming

F-box and ubiquitin-mediated proteolysis are regulators of key cellular processes including signal transduction, cell cycle, and stress responses (biotic and abiotic) in plants [26]. In IC-8219 cultivar, 23 (14 induced and 9 repressed) DEGs encoding putative E3 ubiquitin protein-ligase, F-Box protein, ubiquitin-conjugating enzyme E2, protein AMN1 homolog, BTB/POZ domain-containing protein, kelch repeat-containing protein, F-box/kelch-repeat protein exhibited a significant difference in expression due to bruchid oviposition (Additional file 6: Table S6). However, in the T-9 cultivar only 4 DEGs were differentially expressed (Additional file 6: Table S7).

Protein kinases/phosphatases also play an important role in responding to various stress signals. We found 37 differentially expressed genes of protein kinases/phosphatases in the IC-8219 due to bruchid egg-laying. About 25 kinases/phosphatases were induced, while 12 were repressed in IC-8219. A large proportion of the DEGs encoding serine/threonine-protein kinase (7 induced) followed by protein phosphatase 2C (3 induced and 1 repressed), receptor-like protein kinase (2 induced and 2 repressed), probable tyrosine-protein phosphatase (3 induced), and a CBL-interacting serine/threonine-protein kinase (Additional file 6: Table S6) were expressed in IC-8219. Mitogen-activated protein kinase kinase 3 (MAPKK3) (XP_014503615.1), which is an important gene of kinase signaling pathways, participating in resistance/susceptibility reaction in most of the crop species was found to be down-regulated in the IC-8219 cultivar. In the T-9 cultivar, we found less representation of DEGs associated with signal transduction pathways (Additional file 6: Table S6).

We also found the up-regulation of 4 Golgi-localized Ca^2+^/cation antiporter genes in the IC-8219 cultivar in response to bruchid oviposition, which might be associated with the cytosolic Ca^2+^ influx during oxidative stress (Additional file 6: Table S6). In the T-9 cultivar, only 3 Ca^2+^ signaling associated genes showed up-regulation (Additional file 6: Table S6).

### Identification of DEGs related to phytohormone signaling

Phytohormone induces a variety of responses in plants against insect oviposition. In the IC-8219, 8 DEGs (2 induced and 6 repressed) associated with auxin signaling pathway was recorded, including indole-3-acetic acid-induced protein ARG2 (1 induced), auxin-responsive protein IAA29 (1 induced), ABSCISIC ACID-INSENSITIVE 5-like protein (2 repressed), 1-aminocyclopropane-1-carboxylate oxidase (1 repressed), indole-3-acetic acid-amido synthetase (1 repressed) and auxin-responsive protein (1 repressed) (Additional file 6: Table S6). However, only 4 phytohormone related genes that are involved in the ABA signaling pathway expressed in the T-9 cultivar. The indole-3-acetic acid-induced protein ARG2 (P32292.1) was shared by both the cultivars (Additional file S6: Table S6).

### Differential expression of genes associated with metabolism and biosynthesis

In response to bruchid oviposition, plants may alter many of their metabolic pathways. Based on GO and KEGG pathway enrichment analyses, the IC-8219 cultivar showed differential expression of 29 and 16 DEGs associated with carbohydrate metabolism and lipid metabolism, respectively. Interestingly, the majority (28) of DEGs were repressed in oviposited pods of IC-8219 (Additional file 7: Table S7). A similar (14 down-regulated) trend was observed for DEGs of lipid metabolism in IC-8219 (Additional file 8: Table S8). In the case of cultivar T-9, only two DEGs of carbohydrate metabolism were significantly repressed (Additional file 9: Table S9), while no lipid metabolism DEGs were represented.

Furthermore, GO and KEGG pathway enrichment analyses revealed the up-regulation of several genes associated with secondary metabolic pathways, including isoflavonoid biosynthesis (ko00943) and tropane, piperidine, and pyridine alkaloid biosynthesis (ko00960). Evidently, 12 genes (9 induced and 3 repressed) related to secondary metabolite production were found to be differentially expressed in the IC-8219 cultivar (Additional file 6: Table S6). Whereas, in the T-9 cultivar 7 genes under this category showed significant up-regulation (Additional file 6: Table S6).

### Validation of the expression of DEGs by qPCR

**Fig. 5:**
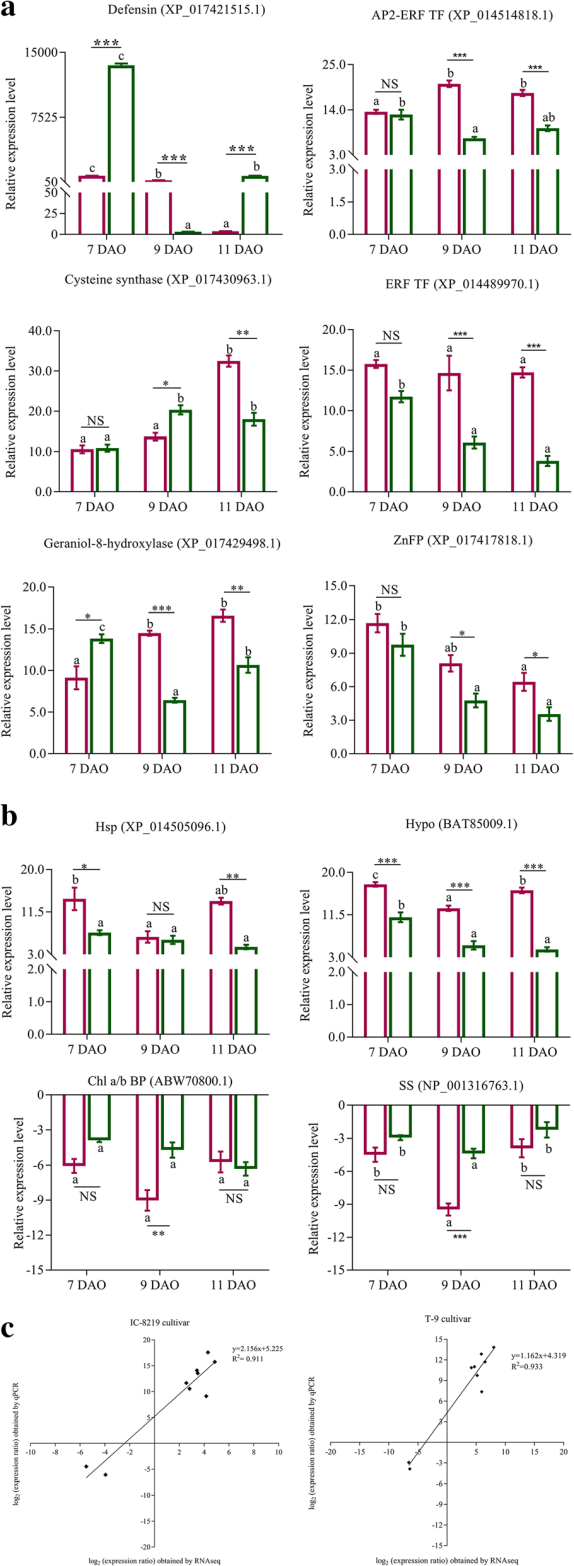
Levels of expression of black gram genes in response to bruchid ovipositioning at various time intervals. **a** Expression of 8 up-regulated genes. **b** Expression of 2 down-regulated genes. The levels of expression of each gene were normalized using the *EF* 1α as an endogenous gene. The y-axis indicates the fold change increase/decrease in the expression level of the genes. **c** Correlation analysis of log_2_ folds change values obtained by RNAseq and qPCR in the IC-8219 and T-9 cultivars. Bars represent Mean ± SEM. The different alphabets indicate comparison within the group (*P*-value < 0.05) and the asterisk and NS indicate comparison between the group. * P-value < 0.05, ** P-value < 0.01, *** P-value < 0.0001 and NS = P-value > 0.05. The pink color bars indicate IC-8219 (T) samples and the green color bars indicate T-9 (T) samples

To validate the transcriptome data, we quantified relative expression levels of 10 (8 up- and 2 down-regulated) selected genes represented in both the cultivars by quantitative PCR (qPCR) analysis using primers listed in Additional file 10, Table S10. These were genes encoding defensin (XP_017421515.1), AP2 ethylene-responsive transcription factor (XP_014514818.1), IAA induced protein (P32292.1), cysteine synthase (XP_017430963.1), geraniol-8-hydroxylase (XP_-17,429,498.1), ethylene-responsive transcription factor (XP_014489970.1), zinc finger protein (XP_017417818.1), HSP (XP_014505096.1), hypothetical protein (BAT85009.1), Chlorophyll a/b binding protein (ABW70800.1) and sucrose synthase (NP_001316763.1). The gene elongation factor 1α (EF 1α) was used as an internal control. The temporal expression pattern of the selected genes to understand the relative abundance in both the cultivars was studied. The relative expression pattern of the defensin gene was induced by oviposition or expressed due to the activation of transcription factors such as the AP2-like ethylene-responsive transcription factor was also measured. The qPCR analyses showed that the differentially expressed genes followed a concordant direction of fold change as revealed by RNA seq data (Fig. 5a and b). Furthermore, linear regression analyses showed a positive correlation between the qPCR and RNA-seq data, which confirmed that RNA-seq data were consistent or reliable (Fig. 5c).

### Quantitative estimation of anti-digestive enzymes

**Fig. 6:**
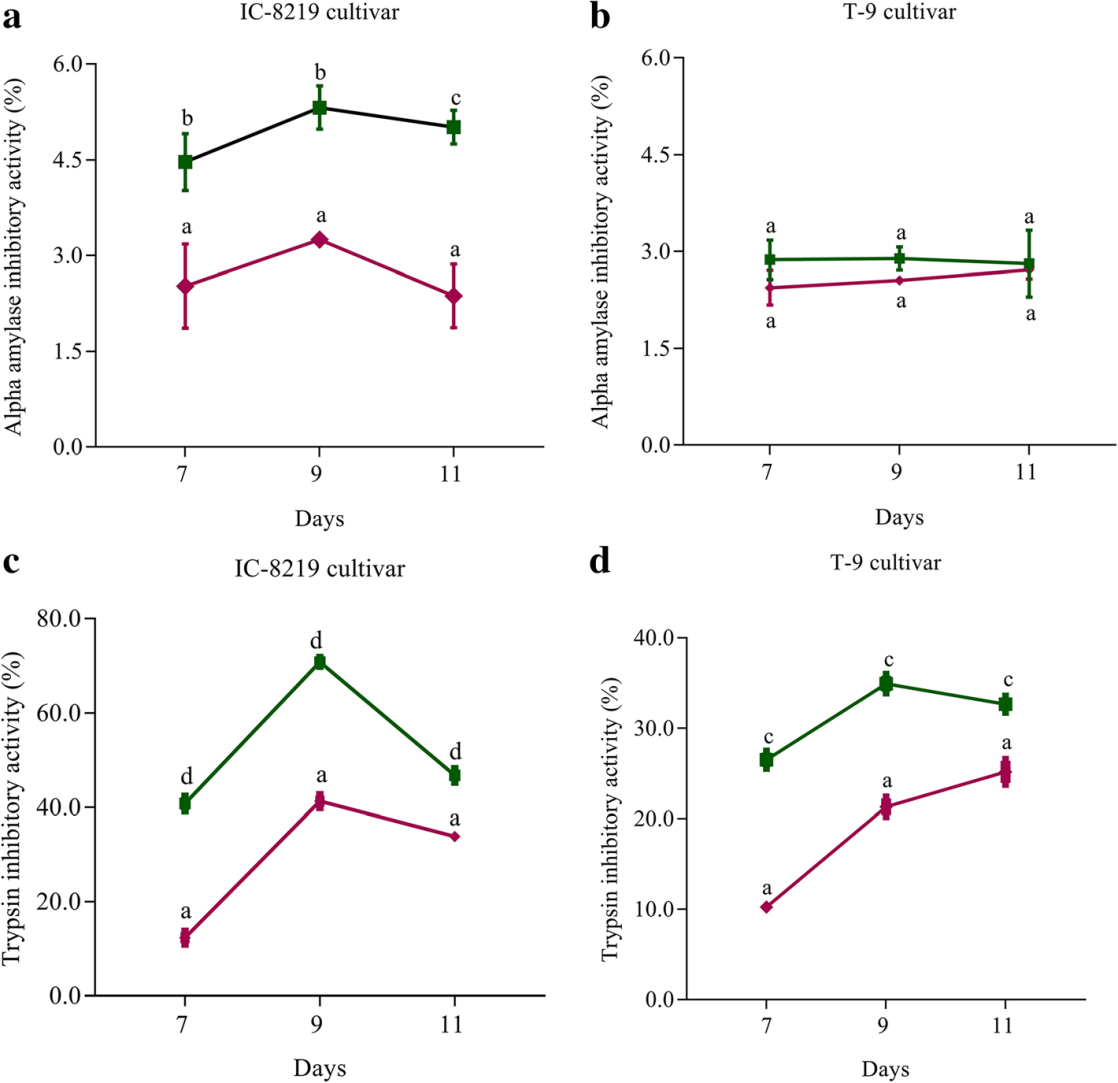
Activity of enzyme inhibitors in black gram due to bruchid beetle ovipositioning*.* **a** α-AI activity in the treated and control samples of IC-8219. **b** α-AI in the treated and control samples of T-9. **c** TI activity assay in the treated and control samples of IC-8219. **d** TI activity assay in the treated and control samples of T-9. The bars represent Mean ± SEM. The different alphabets above the bars indicate comparison within the group (P-value < 0.05). The pink color line indicates data of control samples and the green color line indicates data of treated samples

Accumulation of digestive enzyme inhibitor proteins such as α-amylase inhibitor (α-AI) and trypsin inhibitor (TI) are predominantly induced upon insect herbivory [27]. Therefore, we quantified the accumulation of α-AI and TI in the immature seeds of bruchid oviposited pods of both the cultivars. The activity of both α-AI and TI were higher in the bruchid oviposited pods compared to the controls in both the cultivars. The α-AI activity in IC-8219 was significantly higher up to 11 days than T-9 (Fig. 6a). The α-AI activity in bruchid-oviposited T-9 cultivar was significantly higher when compared with their controls (Fig. 6b). Although TI-activity was significantly higher in the bruchid-oviposited pods of both the cultivars as compared to their controls, however, in IC-8219 TI activity was highest (> 70%) after 9 days of oviposition compared to controls and T-9 (Fig. 6c and d).

## Discussion

Eggs represent a future threat to a host plant and thus the pre-activation of defenses by a host plant is an advantage if they can anticipate the upcoming damage. Both direct and indirect responses to oviposition have been observed in plants [1, 28]. However, there is limited information on the molecular changes that take place in the host due to egg laying by female insects. We studied the response of immature black gram pods upon ovipositioning by bruchids and analyzed comparative transcript profiles of pods of a moderately resistant (IC-8219) and susceptible (T-9) cultivars. The result of transcriptome analysis revealed that ovipositioning triggered the activation of many defense-related genes in both moderately resistant and susceptible cultivars. In our previous study on transcript dynamics in bruchid-oviposited black gram pods of IC-8219 cultivar, we got the clues that ovipositioning triggers up-regulation of many defense-related genes such as defensin, PR, lipoxygenase, signal transduction pathway genes [20]. The present study provided comprehensive data on transcript changes in IC-8219 and T-9 cultivars of black gram. The major functional categories of genes that were differentially expressed are grouped under defense-related genes, oxidative stress, production of secondary metabolites, signal transduction pathways, and several other defense responsive genes. Similar responses were found in *Arabidopsis* due to pierid butterfly oviposition [12] and ovipositioning by *Xanthogaleruca luteola* in elm [29]*.*

### Role of ROS accumulation in bruchid defense

The earliest evidence of a defensive response by a plant is the production of ROS as a part of the HR response. Oviposition triggers local ROS accumulation and cell death mostly mediated by the salicylic acid (SA) pathway [12, 13]. We found ROS accumulation in oviposited pods of both the black gram cultivars was due to the bruchids and could be involved in the defense response by activating the systemic acquired resistance [30, 31]. In *Solanum dulcamara,* the accumulation of H_2_O_2_ was found at the oviposition sites by *Spodoptera exigua*. A similar response was observed in *Arabidopsis thaliana*, tomato, and pine, but only the last species showed an obvious HR-like response to insect oviposition [12–14]. The accumulation of ROS leads to an increase in the phytohormone, SA, and the induction of SA-responsive genes [12, 13]. In black gram pods, we found a plethora of SA pathway-related genes suggesting the HRs were elicited by bruchid oviposition.

### Bruchid oviposition induced defense-related genes

A potent mediator of plant defense gene expression against insects is salicylic acid (SA), [32]. It has been reported that SA accumulates at high levels in response to insect eggs, indicating the involvement of the SA pathway in response to ovipositioning [33–36]. We observed expression of DEGs related to PR proteins such as PR2, glucan endo-1,3-beta-glucosidase, thaumatin (PR5), and pathogen-related protein after bruchid egg-laying. The four PR related genes [Pathogen related protein 2-like (XP_014509125.1), glucan endo-1, 3-β-glucosidase (XP_014502683.1), thaumatin-like protein (XP_014520780.1), and pathogen-related protein (XP_017421074.1)] were significantly induced in IC-8219 in response to oviposition, while only one PR related gene [Endochitinase PR4 (XP_014492850.1)] was up-regulated in the T-9 cultivar. The SA responsive gene PR-1 was induced by oviposition in black gram as observed in other crops [5, 12] and its expression was not detected in SA-signaling *Arabidopsis* mutants eds1–2, sid2–1, and npr1–1 [13]. In *Arabidopsis*, ovipositioning by *P. brassicae* triggered accumulation and expression of SA-responsive genes [12] and indicated that a functional SA pathway is required for egg-laying induced expression of defense genes, ROS accumulation, and local cell death [13].

We also found a very small representation of jasmonic acid (JA) mediated genes, which could be due to the suppression of the JA signaling pathway by SA. The down-regulation of 3 oxylipin biosynthetic genes in IC-8219 suggested that ovipositioning mediated defense response was limited to a distinct subset of numerous JA-mediated defense traits [12]. The activation of the SA pathway is known to act antagonistically to JA signaling pathway in plants, however, the mechanism is yet to be elucidated. Significant progress has been made in *Arabidopsis* to identify specific targets of SA in the JA pathway by which it exerts an antagonistic effect [37, 38].

Plant protease inhibitors (PIs) constitute one of the most important plant defensive traits against insect pests [39, 40]. These proteases perform different functions like initiating activation of various signaling processes, releasing signal peptides, and finally regulating various signal pathways of effectors triggered immunity, systemic acquired resistance, and induced systemic resistance. There are three main subclasses of proteases involved in arthropod digestion, serine-, cysteine-, and aspartic-proteases, grouped according to the reactive amino acid of their active site group [41]. We found several DEGs having aspartic-protease endopeptidase activity were induced in IC-8219 upon bruchid ovipositioning when compared to T-9. The expression of enzyme inhibitors in IC-8219 could be an effective mode of protection from bruchid infestation. The over-expression of DEGs having aspartic-endopeptidase activity is mediated by SA, JA, and ethylene (ET). To reduce defense-related fitness costs, plant steers their induced defense responses by cross-communicating between SA and JA signaling pathways was reported by various groups [37, 42, 43].

The majority of the defense responsive genes in the IC-8219 cultivar were pentatricopeptide repeat-containing proteins (PPRs) suggesting that post-transcriptional modification could be an important feature in the bruchid ovipositioning-mediated HR in black gram. PPRs are reportedly involved in a wide range of different post-transcriptional processes in plant organelles [44–46] and may also affect nuclear gene expression by retrograde signaling [47]. Moreover, recent research revealed that PPRs are also involved in electron transport, reactive oxygen species generation, and abiotic stress resistance [48]. In *Arabidopsis thaliana,* PPR protein family genes have been implicated in *Arabidopsis* defense response against biotic and abiotic stress. For example, a member of the PPR protein family, the MEF11/LOI1 gene, is involved in mitochondrial RNA editing and biosynthesis of secondary metabolites (isoprenoids) in response to wounding and pathogen attack in *Arabidopsis* [49, 50]. Park and his co-worker [51] demonstrated that miRNA400 guided suppression of PPR1 and PPR2 protein in *Arabidopsis* renders the plant more vulnerable to bacterial and fungal pathogen attack. Thus, PPRs in black gram appeared to be involved in defense against bruchid ovipositioning.

In the IC-8219 cultivar, we also found differential expression of nudix hydrolases, which catalyzes the hydrolysis of nucleoside diphosphates such as nucleotide sugars (ADP-glucose) [52–54] and pyridine nucleotides such as NADH, NADPH, and 8-oxo-GTP [55, 56]. Nucleoside diphosphates are major metabolic intermediates and signaling molecules, which are often toxic to the cell. Therefore, nudix hydrolases in bruchid oviposited black gram pods might be involved in maintaining cellular homeostasis by detoxifying the excess nucleotide diphosphates. Dysfunction of these house cleaning and oxidation protective enzymes causes disruption of cellular homeostasis which could severely affect the pathogen defense and hormone signaling pathways in plants [57].

### Role of transcription factors in bruchid egg induced defense

The expression of defense responsive genes is mediated by activation of transcription factors; hence the identification of such TFs is critical for defense response. However, signaling molecules play an important role in TFs expression; for example, SA suppresses the JA pathway downstream of JA biosynthesis and the JAZ–COI1 complex TFs as well as expression of several TFs [58, 59]. We found that bruchid oviposition in both the cultivars activated several ethylene-responsive factors (ERFs), including AP2-like ERF (APETALA2/Ethylene Responsive Transcription Factor) genes. The GCC-box is a binding site for members of the AP2/ERF TF, including transcription factors ERF and ORA59, which activates plant defensin gene *PDF1.2* [60, 61]. SA has been found to be negatively regulating TF ORA59 but not that of ERF1 indicating the antagonistic effect of SA on JA-responsive *PDF1*.*2* gene expression [59, 62, 63]. Similar crosstalk could be present in black gram during interactions with bruchid ovipositioning.

Production of nitric oxide (NO) is a well-known fact during insect herbivory [64]. In the IC-8219 cultivar, 5 DEGs under the transcription factor belonged to zinc finger TF SRG1. SRG1 is a positive regulator of nitric oxide (NO) bioactivity during plant immunity [65]. NO is a gaseous lipophilic free radical and key signaling molecule in plants. Over-expression of these TFs in IC-8219 could be associated with the enhanced level of resistance as compared to T-9.

Transcription factors, NAC and MYB, are involved in the plant defense responses against insect herbivory [66] and these TFs were abundant in IC-8219. Both NAC and Zn finger TFs are induced upon *Spodoptera littoralis* feeding in *Arabidopsis* [67]. SA mediated induction of MYB TFs have been found to play an important role in defense responses in *Arabidopsis* [68]**.** These MYB TFs in black gram could be responsible for regulating various steps of the phenylpropanoid pathway during bruchid ovipositioning.

### Importance of phenylpropanoid and antioxidative enzymes

Secondary metabolite production is crucial for the plants, as it contributes to both inducible and constitutive plant defense response against a variety of insect herbivores, pathogens, and other competitors [69, 70] and attracts parasitoids [71]. Phenylpropanoid is one of the crucial secondary metabolites producing pathways in plants, which is found to be positively related to increased plant resistance to insect herbivores and plant pathogens [72, 73]. The up-regulation of numerous genes of the phenylpropanoid pathway in the IC-8219 cultivar suggested that these genes are important for resistance against bruchid ovipositioning. Thus the production of secondary metabolites appeared to be a common defense mechanism in black gram against bruchid oviposition.

The oxidative status of the plant is related to host plant resistance (HPR) to numerous herbivores and pathogens [74], which results in the generation of ROS that is detoxified by different antioxidative enzymes. In all, 16 DEGs having antioxidative activity were enriched in IC-8219 in response to bruchid ovipositioning of which CBS domain-containing protein (CDCPs) accounted for the major proportion. These CDCPs appeared to play an important role in the regulation of many antioxidative enzymes and thereby contributing to the maintenance of intracellular redox balance in black gram during bruchid ovipositioning as observed in *Arabidopsis* and rice [75].

The cytochrome P450 (CYP) superfamily promotes plant growth and development as well as protects plants from various stresses through the manipulation of numerous biosynthetic and detoxification pathways [76]. Both IC-8219 and T-9 cultivars showed differential expression of 6 and 1 DEGs, respectively, under this category indicating that CYP450s could be involved in the biosynthesis of defense compounds such as phenolics and their conjugates, flavonoids, coumarins, lignans, glucosinolates, cyanogenic glucosides, benzoxazinones, isoprenoids, alkaloids. In chickpea, a simulated herbivore by *Helicoverpa armigera* study revealed the induction of several genes of the CYP450 family [77].

We also observed differential expression of sHSPs in both IC-8219 and T-9 due to ovipositioning by bruchid beetles. These sHSPs appeared to have a distinctive role in the induction of HR-independent defense response in black gram as observed in *Nicotiana* against biotic stresses [78].

### Influence of signal transduction pathway on bruchid oviposition response

Plants recognize herbivore-associated molecular patterns (HAMPs) which often rely on receptors implicated in elicitor recognition commonly known as cell surface pattern recognition receptors (PRRs) interactions [36] including receptor-like kinases (RLKs) [79]. Interestingly, 4 RLKs were up-regulated in black gram, indicating that these RLKs could be acting as the first line of defense in responses to oviposition. *Arabidopsis* RLKs, lectin receptor kinase (LecRK-I.8a), involved in the recognition of *Pieris brassicae* egg was an L-(legume) type LecRK [13, 34].

Calcium plays an essential role in the signaling network of plant cells and in regulating plant responses to insects. In IC-8219, we identified Calcineurin B-like proteins (CBL)-interacting serine/threonine-protein kinase, which is a calcium-sensing kinase. Moreover, significant induction of genes encoding yeast GDT1 (GCR1 DEPENDENT TRANSLATION FACTOR 1), a Golgi localized Ca^2+^/H^+^ antiporter [80] sharing homology to *Arabidopsis* (AtGDT-Like2) [81] and these kinases could be contributing to calcium homeostasis in cells in the IC-8219 cultivar. Thus, changes in the intracellular calcium concentration might have activated CBL-interacting serine/threonine-protein kinase in black gram. Recently, soybean receptor-like kinases were found to perceive signals associated with herbivory danger signals (HDSs) in soybean and Arabidopsis [82].

Mitogen-activated protein kinases (MAPK) are important regulatory proteins involved in signal transduction due to insect herbivory [83]. MAPK genes were found to be down-regulated in IC-8219. We found 3 DEGs of protein tyrosine-protein phosphatase, which might be responsible for negative feedback inhibition of MAPK genes [84].

### Role of auxin in black gram defense against bruchid oviposition

In our study, bruchid oviposition did not significantly trigger the induction of various phytohormone related genes in both the cultivars except for indole-3-acetic acid (IAA)-induced protein ARG2 and auxin-responsive protein IAA29 in both IC-8219 and T-9. Moreover, in T-9 ABA-mediated signaling genes (zeaxanthin epoxidase, abscisate beta-glucosyltransferase) were also identified. The up-regulation of phytohormone genes in both the cultivars could be involved in cell division and neoplasm formation at the egg-laden site to dislodge eggs from the surface and impede the entry of hatched larvae [3, 85, 86].

### Down-regulation of primary metabolites

Several genes involved in cell wall metabolism, carbohydrate metabolism, and lipid metabolism were down-regulated due to bruchid oviposition on black gram pods. Down-regulation of primary metabolic processes in response to ovipositioning in black gram suggested that the energy is diverted to protect from bruchid infestation. The down-regulation of the above genes was found in *Arabidopsis* in response to ovipositioning by pierid butterflies [12].

### Role of proteins inhibitors during oviposition

Many insecticidal proteins and molecules originating from plants can retard insect growth and development following ingestion, including α-AIs [87]. The α-AIs are not only found to be involved in the impairment of bruchid digestive enzymes but also can act as a biocontrol agent against them [88]. Plants utilize them against a variety of herbivorous insects belonging to Lepidopteran, Coleopteran, Diptera, and Homoptera [89, 90].

Accumulation of protease inhibitors (PIs) such as trypsin inhibitors (TIs) also interferes with normal physiological processes of the insect gut [91]. TIs mostly inhibit the digestion of proteins and thereby results in the deficiency of essential amino acids, developmental delay, mortality, and/or reduced fecundity [92]. Higher TI-activity in IC-8219 as compared to the T-9 cultivar could be an important characteristic of IC-8219 for moderate resistance against bruchids. High levels of accumulation of trypsin inhibitors have been found in many bruchid-resistant varieties of mungbean [93].

## Conclusion

**Fig. 7:**
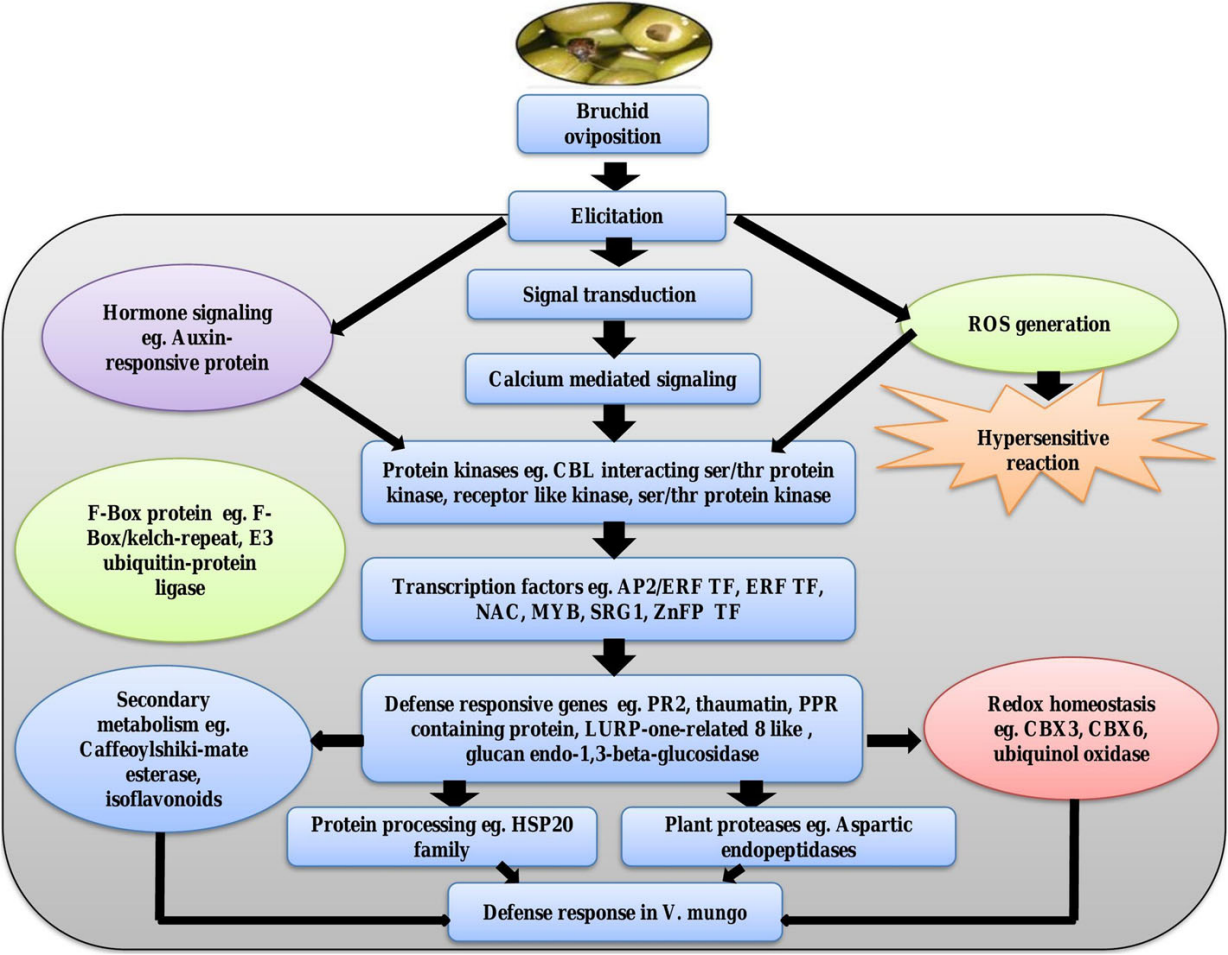
Hypothetical diagram showing the molecular events occurring in *V. mungo* pods in response to bruchid ovipositioning

We studied the transcript dynamics of black gram pods against bruchid oviposition in a moderately resistant (IC-8219) and a susceptible cultivar (T-9) by comparative transcriptome sequencing. Our data revealed an interesting finding that the black gram pods respond to the initial egg-laying and quickly reset their perception and signal transduction system and prepare for the damage by the hatched larvae. Most of the DEGs related to signaling and downstream defense were up-regulated in both IC-8219 and T-9 with significant differences. The bruchid egg laying might be related to the changes in functions and metabolic pathways of some key DEGs, such as those involved in the ROS removal system, phytohormone signaling pathways, mainly the SA pathway. Interestingly, SA antagonizes the JA-mediated pathway by down-regulating them. Based on transcriptomic data a hypothetical diagram of the response to bruchid oviposition in black gram is shown in Fig. 7. These results will help elucidate the molecular mechanism of response to bruchid egg-laying in black gram and provide a valuable resource of black gram defense genes. A future challenge for this research would be to study SA/JA cross-talk and identify how SA is interacting with JA signaling to suppress JA-dependent gene transcription as observed in the current study. Also, the overexpression *defensin* gene in black gram for bruchid resistance would be interesting to understand the level of resistance.

The results of the current study are concurrent with a recent report on the identification of QTL controlling bruchid resistance in *V. radiata* [94]. It was found that a major QTL containing various candidate genes such as R-gene families and TFs are responsible for bruchid defense in black gram. Some of these genes, ERFs, plant encoding receptors (STPK), Zn finger proteins, F-box proteins are also represented in our transcriptome profile, which further substantiated our data.

## Methods

### Experimental plant and insects

The seeds of two cultivars (IC-8219 and T-9) of black gram were obtained from the Indian Institute of Pulses Research (IIPR), Kanpur, and sown in the greenhouse. *Callosobruchus spp* were maintained on dried black gram seeds in a plastic container in the laboratory at 25 °C and 65–70% relative humidity (RH). Plants were raised to maturity in greenhouse conditions. Bruchid adults (5 males and 10 females) were released on the pods covered with a muslin cloth in the greenhouse for oviposition. Samples were collected after 7 days of the release of insects or once we saw the eggs deposited on the pod wall. The eggs laid by adult females on mature pods takes about 6–7 days to hatch [95].

### DAB assay

Bruchid non-oviposited (Control; C) and oviposited (Treated; T) pods of black gram collected from both the cultivar after 7 days oviposition (DAO) and dipped in 3,3″ Diaminobenzidine (DAB) solution (1 mg/ml; pH 3.6) for overnight at 37 °C by as described in [96].

### RNA extraction and quality control

Total RNA was extracted from two biological replicates of non-oviposited (C) and oviposited (T) plants of both the cultivars collected at 7 days. The RNA extracted from the developing seeds collected after 7 days of oviposition from IC-8219 and T-9 cultivar were used for RNAseq analysis. The total RNA was extracted using the PureLink® Plant RNA reagent (Ambion) kit following the manufacturer’s instructions. The quality and quantity of the extracted RNA samples were checked on 1% denaturing RNA agarose gel and Nanodrop/Qubit Fluorometer, respectively. The RNA samples with a 260/280 ratio ranging from 1.8–2.0 and a 260/230 ratio from 2.0–2.4 were used for further analysis. These RNA samples were finally used for Illumina sequencing and gene expression analysis.

### Illumina sequencing and data processing

In all, eight RNAseq paired-end libraries were prepared from total RNA samples using the IlluminaTruSeq stranded mRNA sample preparation kit. The cDNA libraries were generated using mRNA enriched from the total RNA using poly-T attached magnetic beads, followed by enzymatic fragmentation and 1st strand cDNA conversion using a SuperScript II and Act-D mix to facilitate RNA dependent synthesis. The 1st strand cDNAs were synthesized to the 2nd strand using the 2nd strand mix. The ds cDNAs were purified AMPure XP beads followed by A-tailing, adapter ligation, and enrichment by a limited number of PCR cycles. The PCR enriched libraries were analyzed in a 4200 Tap Station system (Agilent Technologies) using high sensitivity D5000 screen tape as per the manufacturer’s instructions. The paired-end libraries were sequenced on the NextSeq 500 (2 × 150 bp) to generate libraries of sizes ranging between 408 bp and 475 bp for all eight samples. The sequenced raw data from IC-8219 (C), IC-8219 (T), T-9 (C), and T-9 (T) (2 biological replicates from each sample) were processed to obtain high-quality concordant reads using Trimmomatic v0.35 (http://www.usadellab.org/cms/index.php?page=trimmomatic) and adapter sequences, ambiguous reads (reads with unknown nucleotides “N” larger than 5%) and low-quality sequences (reads with more than 10% quality threshold (QV) < 20 Phred score) were removed by an in-house script. The resulting high quality (QV > 20), paired-end reads were used for the de novo assembly. The above work was carried out using the services of the Xeleris Technology Service, Ahmadabad, Gujarat.

### De novo transcriptome analysis, functional annotation and differential gene expression (DGE)

The filtered high-quality reads of all the samples were pooled together and assembled into transcripts using Trinity de novo assembler (V2.5) (http://trinityrnaseq.github.io) [97] with a fixed default K-mer size of 25 and minimum contig length 200. The assembled transcripts were then further clustered together using the CD-HIT-EST-4.5.4 (http://www.bioinformatics.org/downloads/index.php/cd-hit/cd-hit-v4.5.4/) software to remove the isoform produced during assembly to generate unigenes. Only those unigenes which were found to have > 80% coverage and 3X read depth were considered for downstream analyses. The TransDecoder-v2.0 was used to predict coding sequences (CDSs) from the generated unigenes.

Functional annotation of the CDS sequences was performed using the DIAMOND program (BLASTx mode) DIAMOND (BLASTx mode) (https://github.com/bbuchfink/diamond/) which help find the homologous sequences for the genes against NR (http://www.ncbi.nlm.nih.gov/) from the NCBI. To identify CDSs for each of the four samples from a pooled set of CDSs reads from each of the samples were mapped to the final set of pooled CDSs using the bwa (−mem) toolkit. The read count (RC) values were calculated from the resulting mapping and those CDSs having > 80% coverage and 3X read depth were considered for differential expression analysis. The negative binomial distribution model of the DEseq package (version 1.22-http//www.huber.embl.de/users/anders/DEseq/) was also used to calculate differential gene expression. The CDSs with fold change (FC) values greater than two were considered as up-regulated, whereas less than two were considered down-regulated. Genes with FDR value ≤0.05 were considered as significant.

### Gene ontology (GO) and biological pathway analysis

Blast2Go program (Blast2GOPRO) [98] was used to assign the GO terms and to annotate the differentially expressed genes, which allowed them to group under three major categories i.e. biological process, molecular function, and cellular component. Later, the KAAS (Kyoto Encyclopedia of Genes and Genomes Automatic Annotation Server: http://www.genome.jp/keg/kaas/) was used to predict the pathway(s) of the differentially expressed genes. The BBH (Bi-directional best hit) [99] option of the KAAS automated server was used to assign the KEGG orthology (KO) terms.

### Quantitative PCR (qPCR) validation

Total RNA was extracted from three biological replicates of non-oviposited (C) and oviposited (T) plants of both the cultivars and samples were collected at 7, 9, and 11 days after oviposition. The gene-specific primers were designed using the Oligo Perfect Designer software program (http://www.thermofisher.com/oligoperfect.html/) having GC content of 55–60%, a T_m_ > 50 °C, primer length ranging from 18 to 22 nucleotides, and expected product size of 100-215 bp. The list of primers used for qPCR along with their target genes is listed in Additional file 10, Table S10. Total RNA was extracted from oviposited and non-oviposited samples of both the cultivars using the PureLink™ Plant RNA reagent (Ambion). The cDNA was synthesized using the PrimeScript™ RT reagent Kit with gDNA Eraser (Clontech, USA), and the real-time PCR protocol was followed according to the manufacturer’s instructions given in the SYBR® PremixExTaq™ (Tli RNase H Plus) (Clontech, USA). The temperature profile used in the Applied Biosystems StepOnePlus™ Real-Time PCR System (Applied Biosystems, USA) was 95 °C for 30 s., 40 cycles of 95 °C for 5 s. and 60 °C for 30 s. followed by a melt curve stage at 60 °C for 1 min. The primer for the elongation factor (EF-1α) gene was used as an endogenous control. Tissue samples were used for qPCR analyses and the 2^-∆∆^CT method was used to deduce the relative quantification (RQ) value of each sample based on normalization with the reference gene. The PCR analyses were done thrice using two biological replicates and each reaction was run in triplicate using the designed gene-specific primers. The mean RQ values were used for analyses.

### Amylase (α-AI) and trypsin inhibitor (TI) assays

The total proteins from the immature seeds of oviposited (T) and non-oviposited (C) plants were extracted using 20 mM phosphate buffer (pH 6.9). For TI activity, crude protein (50 μl) was mixed with 20 μl bovine pancreas trypsin (1 mg ml^− 1^) and incubated following the procedure described in Nair et al. [100].

The α-AI activity was assessed by quantifying the reducing sugar following the protocol of [101]. In brief, an α-Amylase enzyme and crude protein followed by the addition of starch and dinitrosalicylic acid (DNSA) reagent according to the protocol described in Gupta et al. [101].

### Statistical analyses

The data were expressed as mean ± SEM. The differences within a group and between the groups were assessed by within-group and between groups mixed ANOVA. The post-hoc analysis was performed by the Bonferroni method at the 95% confidence level. The analysis was performed in statistical software R [102].

## Supplementary Information

**Additional file 1: Table S1**: KEGG pathway annotation of 4322 CDSs of IC-8219 cultivar and 3551 CDSs of T-9 cultivar.

**Additional file 2: Table S2**: Total number of CDSs of IC-8219 (C), IC-8219 (T), T-9 (C), and T-9 (T) samples under 23 identified KEGG pathway

**Additional file 3: Table S3**: 630 DEGs in IC-8219 cultivar and 184 DEGs in T-9 cultivar

**Additional file 4: Table S4**: Significantly enriched GO terms of IC-8219 and T-9 cultivar

**Additional file 5: Table S5**: Significantly enriched KEGG pathways in both IC-8219 and T-9 cultivars

**Additional file 6: Table S6**: List of DEGs in the T-9 and IC-8219 cultivars under a different category

**Additional file 7: Table S7**: 29 down-regulated genes of a primary metabolic pathway in the IC-8219 cultivar in response to bruchid oviposition

**Additional file 8:.** Table S8: 16 down-regulated genes of lipid metabolic pathway in the IC-8219 cultivar in response to bruchid oviposition

**Additional file 9: Table S9**: 2 down-regulated primary metabolic genes in the T-9 cultivar in response bruchid oviposition

**Additional file 10: Table S10**: List of qPCR primers used for validation of transcriptome data

## Data Availability

The data set supporting the conclusion of this article are available in the NCBI Sequence Read Archive (SRA) under accession number PRJNA604405 (http://www.ncbi.nlm.nih.gov/sra/PRJNA604405).

## Abbreviations

FDR: False discovery rate
CDSs: Coding sequences
DEGs: Differentially expressed genes
HR: Hypersensitive reaction
ROS: Reactive oxygen species
bp: Base pair
cDNA: Complementary deoxyribonucleic acid
GO: Gene ontology
Nr: Non-redundant protein database
NCBI: National Centre for Biotechnology Information
BLAST: Basic local alignment search tool
KEGG: Kyoto encyclopedia of genes and genomes
TF: Transcription factor
qRT-PCR: Quantitative real-time polymerase chain reaction
mRNA: Messenger ribonucleic acid
mM: Milli mole
α-AI: Alpha-amylase inhibitor
TCA: Trichloroacetic acid
DMSO: Dimethyl sulfoxide
BA*p*NA: N_α_-Benzoyl-DL-arginine p-nitroanilide hydrochloride
^o^C: Degree Celcius
μl: Microlitre
ds cDNA: Double-stranded complementary deoxyribonucleic acid
nm: Nanometer
